# Effectiveness of influenza and pneumococcal polysaccharide vaccines against influenza-related outcomes including pneumonia and acute exacerbation of cardiopulmonary diseases: Analysis by dominant viral subtype and vaccine matching

**DOI:** 10.1371/journal.pone.0207918

**Published:** 2018-12-06

**Authors:** Joon Young Song, Ji Yun Noh, Jin Soo Lee, Seong-Heon Wie, Young Keun Kim, Jacob Lee, Hye Won Jeong, Shin Woo Kim, Sun Hee Lee, Kyung-Hwa Park, Won Suk Choi, Hee Jin Cheong, Woo Joo Kim

**Affiliations:** 1 Division of Infectious Diseases, Department of Internal Medicine, Korea University College of Medicine, Seoul, Korea; 2 Asian Pacific Influenza Institute (APII), Seoul, Korea; 3 Division of Infectious Diseases, Department of Internal Medicine, Inha University School of Medicine, Incheon, Korea; 4 Division of Infectious Diseases, Department of Internal Medicine, Catholic University Medical College, St. Vincent’s Hospital, Suwon, Korea; 5 Division of Infectious Diseases, Department of Internal Medicine, Yonsei University, Wonju College of Medicine, Wonju, Korea; 6 Division of Infectious Diseases, Department of Internal Medicine, Hallym University College of Medicine, Chuncheon, Korea; 7 Division of Infectious Diseases, Chungbuk National University College of Medicine, Cheongju, Korea; 8 Division of Infectious Diseases, Department of Internal Medicine, Kyungpook National University School of Medicine, Daegu, Korea; 9 Division of Infectious Diseases, Department of Internal Medicine, Pusan National University School of Medicine, Pusan, Korea; 10 Division of Infectious Diseases, Department of Internal Medicine, Chonnam National University Medical School, Gwangju, Korea; Universidade de Lisboa Faculdade de Medicina, PORTUGAL

## Abstract

**Background:**

Influenza and pneumonia are leading causes of morbidity and mortality among the elderly. Although vaccination is a main strategy to prevent these infectious diseases, concerns remain with respect to vaccine effectiveness.

**Methods:**

During three influenza seasons (2014–2015, 2015–2016 and 2016–2017), we evaluated the effectiveness of the influenza and pneumococcal vaccines against pneumonia and acute exacerbation of cardiopulmonary diseases among the elderly aged ≥65 years with influenza-like illness (ILI). Demographic and clinical data were collected prospectively.

**Results:**

Among 2,119 enrolled cases, 1,302 (61.4%) and 871 (41.1%) received the influenza vaccine and 23-valent pneumococcal polysaccharide vaccine (PPV23), respectively. During an A/H3N2-dominant season with poor influenza vaccine effectiveness (2014–2015 season), neither the influenza vaccine nor PPV23 showed significant effectiveness against pneumonia or acute exacerbation of cardiopulmonary diseases. During seasons with good influenza vaccine effectiveness (2015–2016 and 2016–2017 seasons), the influenza vaccine was effective in preventing pneumonia, but PPV23 was not. In particular, the influenza vaccine was effective in preventing acute exacerbation of heart diseases (75.0%) during the A/H1N1-dominant 2015–2016 season.

**Conclusion:**

The influenza vaccine was effective in preventing pneumonia only during vaccine-matched seasons with good effectiveness against circulating influenza viruses. In addition, the influenza vaccine was cardio-protective during a vaccine-matched A/H1N1-dominant season.

## Introduction

Although influenza disease burden might vary according to regional climate, population density, and seasonal viral antigenic changes, the clinical impact of influenza epidemics has been high worldwide. In the United States, influenza epidemics are responsible for approximately 20,000 to 40,000 deaths and 114,000 hospitalizations annually [1, 2]. In South Korea, the influenza epidemic during the 2013–2014 season caused an estimated 23,326 hospitalizations and 1,249 deaths [3]. Globally, the estimated mean annual influenza-associated excess mortality rate ranges from 0.1–6.4 per 100 000 individuals for people younger than 65 years, 2.9–44.0 per 100 000 individuals for people aged 65–74 years, and 17.9–223.5 per 100 000 for people aged ≥75 years [4]. Influenza-related hospitalizations and deaths are mainly caused by accompanying complications, including pneumonia, acute exacerbation of underlying cardiopulmonary diseases, rhabdomyolysis, and acute renal failure. Among influenza-related complications, pneumonia is the most common and serious complication, and pneumococcal pneumonia is especially common following influenza infection [5]. As reported previously, there is a 4-fold higher risk of myocardial infarction, cardiovascular hospitalization, or death within 30 days after a pneumonia event, which declines gradually, but remains 1.5-fold higher for up to 10 years [6].

Vaccination offers the most effective way to prevent influenza/pneumonia-related hospitalization and death. Thus, the Korean government has subsidized the influenza vaccine and 23-valent pneumococcal polysaccharide vaccine (PPV23) for the elderly aged ≥65 years since 1997 and 2013, respectively [7, 8]. Currently in the Republic of Korea (ROK), the coverage rates of influenza and pneumococcal vaccination reach around 80% and 60%, respectively, in the elderly 65 years and older [7, 8]. Despite such high uptake rates, there have been concerns with respect to vaccine effectiveness in the elderly population. Moreover, vaccine effectiveness for influenza varies according to the dominant influenza virus subtype and degree of mismatch between the vaccine and circulating virus strains each season. Nevertheless, influenza vaccination is expected to decrease disease severity, even if laboratory-confirmed influenza itself is not prevented. On the other hand, the effectiveness of PPV23 against non-invasive pneumococcal pneumonia remains controversial. In this study, we evaluated the effectiveness of influenza and pneumococcal vaccines against pneumonia and acute exacerbation of cardiopulmonary diseases among the elderly aged ≥65 years with influenza-like illness (ILI).

## Methods

### Study design

During three influenza seasons (2014–2015, 2015–2016, and 2016–2017), a multicenter prospective cohort study was performed at 10 university hospitals that participated in a hospital-based influenza surveillance system (Hospital-based Influenza Morbidity and Mortality, HIMM)[9]. During the study periods (from October 1 to April 30 each season), patients aged ≥65 years with ILI were enrolled when they visited emergency room with symptom onset ≤7 days. A rapid influenza detection test was performed at the bedside, and respiratory specimens were collected prospectively. Nasal/throat swab specimens were transported to the central HIMM laboratory for multiplex respiratory viral polymerase chain reaction testing, and clinical data were obtained using a structured case report form that included demographics, underlying diseases, influenza/pneumococcal vaccination, influenza-related complications, hospitalization, and 30-day fatalities. Vaccination status was verified using immunization registry data from the Korean Centers for Disease Control and Prevention (KCDC); each case was defined as vaccinated if they received influenza/pneumococcal vaccines at least 21 days before the ILI event. The study was approved by the ethics committee of each participating hospital, and written informed consent was obtained from all enrolled subjects.

### Definition

ILI was defined as sudden onset of fever (≥38°C) accompanied by ≥1 respiratory symptoms, including cough, sore throat, or nasal symptoms [9]. Pneumonia was diagnosed if patients fulfilled the following clinical and radiological criteria: (a) acute pulmonary infiltrate consistent with pneumonia evident on chest radiographs and (b) confirmatory findings on clinical examination [10]. Acute exacerbation of cardiopulmonary disease (chronic heart disease and chronic airway disease) was defined as cases who were hospitalized due to sudden worsening of underlying diseases, including congestive heart failure, ischemic heart diseases, chronic obstructive pulmonary disease, and asthma, according to diagnosis reports written by professional medical attendants. Based on the surveillance data, we defined each season as a specific subtype-dominant period if the subtype was present in more than 50% of circulating viruses [11]. Accordingly, both the 2014–2015 and 2016–2017 seasons were classified as an A/H3N2 subtype-dominant period, while the 2015–2016 season was determined as an A/H1N1 subtype-dominant period.

### Statistical analysis

Statistical analyses were performed using SPSS version 15.0 (SPSS Inc., Chicago, IL, USA). Quantitative data were expressed as mean ± standard deviation. Vaccine effectiveness (VE) was estimated by using the case-control method. Test-negative control was used for the laboratory-confirmed influenza, while ILI patients without each complicated outcome was used as matched control for pneumonia, acute exacerbation of chronic airway diseases, acute exacerbation of chronic heart diseases, hospitalization, and fatality (within 30 days), respectively. Multivariable logistic regression was used to estimate the odds ratio for events (laboratory-confirmed influenza, pneumonia development, acute exacerbation of chronic airway diseases, acute exacerbation of chronic heart diseases, hospitalization, and 30-day fatality) in vaccinated versus unvaccinated subjects. Covariates included age, sex, body mass index (BMI), comorbidities and month of illness onset. In multivariable logistic regression model for each event, we included factors with a p ≤ 0.05 in univariate analysis and potential confounders that might modify the VE. In univariate analysis, the significant difference between cases and controls was estimated by the Chi-squared or the Fisher’s exact test for categorical variables and student-t test for quantitative variables. VE was defined as [100 × (1- odds ratio for event in vaccinated versus non-vaccinated persons)].

### Ethics statement

This study was approved by the Institutional Review Board in Korea University Guro Hospital (approval number: KUGH11088), Korea University Ansan Hospital (AS11047), Chungbuk National University Hospital (2011-06-044), Hallym University Kangnam Sacred Heart Hospital (2011-06-50), Inha University Hospital (11–1534), The Catholic University St. Vincent's Hospital (VC11ONME0118), Wonju Severance Christian Hospital (CR311025), Chonnam National University Hospital (CNUH-2012-133), Kyungpook National University Hospital (2012-07-030), and Pusan National University Hospital (1208-010-009). Written informed consent was obtained from the participants or their legal representatives.

## Results

**Fig 1:**
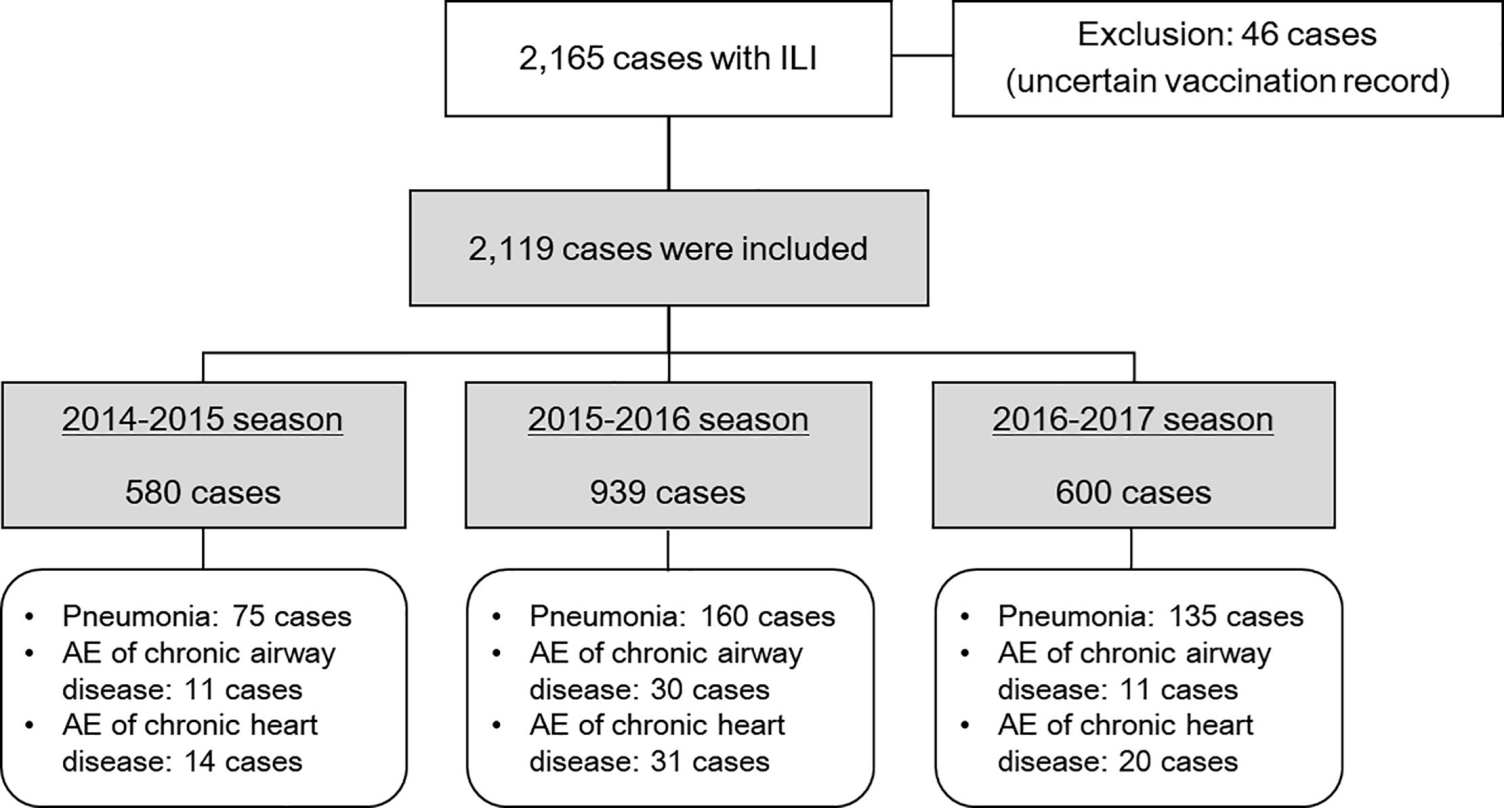
Study flowchart.

During the study periods, 2,165 cases with ILI were registered, of which 46 were excluded because of uncertain vaccination records (Fig 1). The demographic and clinical characteristics of cases with ILI varied by influenza season (Table 1). Among the 2,119 enrolled cases, 1,181 (55.7%) were laboratory-confirmed as having influenza, 880 (41.5%) were hospitalized, and 70 (3.3%) died. Type A influenza infection was predominant across all three influenza seasons (88.2%, 1,042 among 1,181 laboratory-confirmed cases). Among severe cases, 21.8% (463 among 2,119 cases) were accompanied by diverse complications of pneumonia (370, 17.5%), acute exacerbation of chronic heart disease (65, 3.1%) or chronic airway disease (52, 2.5%), acute renal failure (55, 2.6%), rhabdomyolysis (18, 0.8%), encephalitis (2, 0.1%), or myocarditis (2, 0.1%). Forty cases (10.8%) of pneumonia were caused by *Streptococcus pneumoniae*. Among the 2,119 enrolled cases, 1,302 (61.4%) and 871 (41.1%) received the inactivated trivalent influenza vaccine and PPV23, respectively, at least three weeks prior to ILI event (Table 1). Only a small number of patients (3.5%) received the 13-valent pneumococcal conjugate vaccine (PCV13).

**Table 1:** Demographic and clinical characteristics of study subjects.

Characteristics	Total (N = 2,119)	2014–2015 season (N = 580)	2015–2016 season (N = 939)	2016–2017 season (N = 600)
Age (years), mean (SD)	76.0 (6.7)	73.6 (5.0)	76.9 (7.2)	76.5 ± 6.9
Male sex, No. (%)	1,091 (51.5)	300 (51.7)	467 (49.7)	324 (54.0)
Body mass index (kg/m^2^), mean (SD)	22.1 (5.5)	22.4 (5.0)	21.8 (6.4)	22.9 ± 3.7
Lab-confirmed influenza, No. (%)	1,181 (55.7)	305 (52.6)	525 (55.9)	351 (58.5)
Type A	1,042	263	460	319
Type B	139	42	65	32
Current smoking, No. (%)	197 (9.3)	53 (9.1)	80 (8.5)	64 (10.7)
Underlying diseases, No. (%)				
Diabetes mellitus	681 (32.1)	163 (28.1)	309 (32.9)	209 (34.8)
Chronic heart disease	444 (21.0)	88 (15.2)	208 (22.2)	148 (24.7)
Chronic obstructive lung disease	232 (10.9)	53 (9.1)	101 (10.8)	78 (13.0)
Asthma	170 (8.0)	53 (9.1)	71 (7.6)	46 (7.7)
Chronic renal disease	146 (6.9)	28 (4.8)	71 (7.6)	47 (7.8)
Chronic liver disease	55 (2.6)	14 (2.4)	26 (2.8)	15 (2.5)
Cerebrovascular diseases	296 (14.0)	61 (10.5)	132 (14.1)	103 (17.2)
Neuromuscular disease	70 (3.3)	15 (2.6)	35 (3.7)	20 (3.3)
Autoimmune disease	25 (1.2)	5 (0.9)	10 (1.1)	10 (1.7)
Solid cancer	331 (15.6)	87 (15.0)	134 (14.3)	110 (18.3)
Hematologic malignancy	43 (2.0)	13 (2.2)	20 (2.1)	10 (1.7)
Immunosuppressant use	99 (4.7)	19 (3.3)	55 (5.9)	25 (4.2)
LTCF residents, No. (%)	100 (4.7)	17 (2.9)	51 (5.4)	32 (5.3)
Influenza vaccination, No. (%)	1,302 (61.4)	349 (60.2)	476 (50.7)	477 (79.5)
PPV23 vaccination, No. (%)	871 (41.1)	166 (28.6)	311 (33.1)	394 (65.7)
PCV13 vaccination, No. (%)	74 (3.5)	20 (3.4)	27 (2.9)	27 (4.5)
Complications, No. (%)	463 (21.8)	94 (16.2)	212 (22.6)	157 (26.2)
Pneumonia	370 (17.5)	75 (12.9)	160 (17.0)	135 (22.5)
AE of chronic airway disease	52 (2.5)	11 (1.9)	30 (3.2)	11 (1.8)
AE of chronic heart disease	65 (3.1)	14 (2.4)	31 (3.3)	20 (3.3)
Acute renal failure	55 (2.6)	10 (1.7)	36 (3.8)	9 (1.5)
Hospitalization, No. (%)	880 (41.5)	180 (31.0)	394 (42.0)	306 (51.0)
ICU admission, No. (%)	154 (7.3)	32 (5.5)	65 (6.9)	57 (9.5)
30-day mortality, No. (%)	70 (3.3)	16 (2.8)	33 (3.5)	21 (3.5)

LTCF: long-term care facility; PPV23: 23-valent pneumococcal polysaccharide vaccine; PCV13: 13-valent pneumococcal conjugate vaccine; AE: acute exacerbation; ICU: intensive care unit.

During the study periods in South Korea, A/H3N2 subtype influenza was most prevalent in the 2014–2015 and 2016–2017 seasons, while A/H1N1 subtype viruses predominantly circulated in the 2015–2016 season [11]. As for laboratory-confirmed influenza, the adjusted influenza VE estimates are presented in Table 2. During the 2014–2015 season, the influenza vaccine showed no significant effectiveness. In comparison, the overall VE of the seasonal influenza vaccine was statistically significant during the 2015–2016 (31.4%) and 2016–2017 seasons (60.8%). Specifically, VE was 30.3% for influenza A and 44.5% for influenza B during the A/H1N1-dominant 2015–2016 season and 58.3% for influenza A and 73.9% for influenza B during the A/H3N2-dominant 2016–2017 season.

**Table 2:** Influenza vaccine effectiveness for the prevention of laboratory-confirmed influenza.

Study period	Adjusted vaccine effectiveness, % (95% CI)		
	2014–2015 season	2015–2016 season	2016–2017 season
Laboratory-confirmed influenza	-14.3 (-68.0 to 22.2)	31.4 (7.7 to 49.1)	60.8 (33.6 to 76.9)
Influenza A	-12.3 (-66.9 to 24.4)	30.3 (5.6 to 48.6)	58.3 (28.6 to 75.6)
Influenza B	30.3 (-54.6 to 68.6)	44.5 (-0.5 to 70.7)	73.9 (26.0 to 90.8)

As for VE in preventing influenza-related complications, hospitalization, and 30-day mortality, both the influenza vaccine and PPV23 were assessed (Table 3). Overall, seasonal influenza vaccination lowered the risk of pneumonia (35%), acute exacerbation of cardiovascular disease (51%), hospitalization (36%), and 30-day mortality (62%) in the elderly aged ≥65 years, but PPV23 did not. Neither the influenza vaccine nor PPV23 was significantly effective against acute exacerbation of chronic airway disease. These cases were further analyzed according to the dominant influenza viral subtype and influenza VE for laboratory-confirmed influenza (vaccine matching) in each season. During the A/H3N2-dominant season with poor influenza vaccine effectiveness (2014–2015 season), neither influenza vaccination nor PPV23 showed significant effectiveness against pneumonia or acute exacerbation of cardiopulmonary disease. During seasons with good influenza vaccine effectiveness (2015–2016 and 2016–2017), the influenza vaccine was effective in preventing pneumonia, but PPV23 was not (Table 3). In particular, influenza vaccination was effective at preventing acute exacerbation of chronic heart disease (75.0%) and death (81.0%) during the A/H1N1-dominant 2015–2016 season. Crude VE of influenza vaccine and PPV23 are presented in S1, S2, S3 and S4 Tables.

**Table 3:** Adjusted vaccine effectiveness (VE) against pneumonia, acute exacerbation of cardiopulmonary disease, hospitalization, and mortality in patients with influenza-like Illness.

Adjusted VE (95% CI)	Whole study period		A/H3N2-dominant season with poor influenza vaccine effectiveness (2014–2015 season)		A/H1N1-dominant season with good influenza vaccine effectiveness (2015–2016 season)		A/H3N2-dominant season with good influenza vaccine effectiveness (2016–2017 season)	
	Influenza vaccine	PPV23	Influenza vaccine	PPV23	Influenza vaccine	PPV23	Influenza vaccine	PPV23
Pneumonia	35 (14 to 51)	-26 (-66 to 4)	-4 (-83 to 41)	45 (-8 to 71)	42 (12–62)	-52 (-135 to 2)	63 (36 to 78)	-6 (-74 to 35)
AE of chronic airway disease	31 (-39 to 66)	-33 (-170 to 34)	28 (-399 to 89)	56 (-423 to 96)	42 (-45 to 76)	-105 (-419 to 19)	-181 (-4807 to 84)	33 (-284 to 88)
AE of chronic heart disease	51 (9 to 73)	-6 (-98 to 44)	50 (-83 to 86)	-154 (-883 to 34)	75 (27 to 91)	40 (-96 to 82)	-9 (-408 to 77)	-42 (-426 to 62)
Hospitalization	36 (20 to 48)	-9 (-34 to 12)	21 (-19 to 48)	35 (-4 to 60)	41 (20 to 57)	2 (-36 to 30)	63 (38 to 78)	7 (-43 to 40)
30-day mortality	62 (30 to 79)	-29 (-136 to 29)	88 (48 to 97)	-58 (-666 to 67)	81 (46 to 93)	-97 (-430 to 26)	18 (-201 to 78)	16 (-161 to 73)

PPV23: 23-valent pneumococcal polysaccharide vaccine; AE: acute exacerbation

## Discussion

Vaccination is considered as the primary strategy to prevent influenza infection, post-flu pneumonia and other complications. However, the value of influenza vaccination has been questioned with respect to the vaccine mismatch and low efficacy in old adults. Nevertheless, influenza vaccination is highly recommended, and free vaccination has been provided for the elderly aged 65 years and older in South Korea since 1997 [8]. In this study, we evaluated the effectiveness of the influenza vaccine for preventing pneumonia and acute exacerbation of cardiopulmonary disease in older adults across three influenza seasons. We also examined the dominant influenza viral subtype and vaccine match in each season. Consistent with US reports [12], influenza vaccine effectiveness was considerably lower in the 2014–2015 season compared to 2015–2016 and 2016–2017 seasons in South Korea (Table 1). Although expected to lower the risk of post-influenza pneumonia irrespective of vaccine mismatch, the influenza vaccine was only effective against pneumonia during seasons with good VE against laboratory-confirmed influenza. Meta-analyses showed 25–53% effectiveness of the influenza vaccine against pneumonia- or influenza-related hospitalization, but results were not always consistent because of the variable degree of vaccine mismatch during the study periods [13]. Annually, WHO recommends specific vaccine viruses for inclusion in influenza vaccines, but circulating influenza viruses can be geographically diverse in clades/subclades [14, 15]. Thus, the selection of vaccine strains is very important, and each country should refer to regional influenza virus information in deciding the viral composition of influenza vaccines licensed in their country.

In addition to pneumonia, post-influenza hospitalization and mortality are related to acute exacerbation of cardiopulmonary disease. In a US study, approximately 25% of adults with laboratory-confirmed influenza infection had evidence of acute cardiac injury within 30 days, and the majority occurred within three days [16]. Another study reported that the incidence of admission for acute myocardial infarction was six times higher during first seven days after laboratory-confirmed influenza infection compared to the control periods [17]. According to the Cochrane analysis of four secondary prevention trials, influenza vaccination significantly reduced cardiovascular mortality (55%, 95% CI 24–74%)[18]. In another meta-analysis of randomized clinical trials, influenza vaccination reduced major adverse cardiovascular events by 36%, and the effect was greater among the highest-risk patients with more active coronary disease [19]. Likewise, in South Korea, influenza vaccination significantly reduced hospitalization due to new onset or acute exacerbation of ischemic heart disease and congestive heart failure by 56.0–72.6% in patients aged 65 years and older [20, 21]. Influenza/pneumonia is known to induce endothelial dysfunction, hyper-coagulation, and pro-inflammatory cytokine release, which may make patients vulnerable to acute coronary events and exacerbation of heart failure. Influenza vaccination may prevent or mitigate these vicious cascades, thereby reducing acute cardiovascular events [22]. In this study, influenza vaccination significantly lowered acute exacerbation of chronic heart disease during the vaccine-matched A/H1N1 influenza-dominant season, but not in A/H3N2 influenza-dominant seasons. It is unclear whether these inconsistent results are related to viral subtype, the degree of vaccine matching, neuraminidase activity or other virulence factors of circulating influenza viruses. Previously, several studies reported the positive relation between cardiovascular events and increased serum levels of sialic acid and neuraminidase activity [23, 24]. Given the importance of neuraminidase activity, neuraminidase antigen should be better optimized in developing influenza vaccines.

In contrast with its effect on chronic heart diseases, influenza vaccination was not significantly effective in preventing acute exacerbation of chronic airway diseases consistently over the three influenza seasons in this study. However in the Cochrane analysis, influenza vaccination significantly reduced post-flu exacerbation of chronic obstructive pulmonary disease compared with placebo (37%, 95% confidence interval 11 to 64%)[25]. Contrary to the present study, the Cochrane analysis included late exacerbations occurring after three or four weeks. As for asthma, there are few published data, and most cases are limited to children. In a single study with low risk of bias conducted in a pediatric population, influenza vaccination induced no significant reduction in the number, duration, or severity of influenza-related asthma exacerbations [26]. Further studies are required to evaluate influenza VE in preventing early and late exacerbations of chronic airway diseases among adults.

To prevent post-flu pneumonia and cardiovascular complications, pneumococcal vaccination is also recommended for the elderly and patients with chronic illnesses [22, 27]. Despite controversies regarding the effectiveness of PPV23 against non-invasive pneumococcal pneumonia, several recent studies have shown short-term (within three years after vaccination) significant effectiveness (27.4–63.8%) of PPV23 against pneumococcal pneumonia [28–31]. In this study, the proportion of pneumococcal pneumonia was quite low (11%) owing to the herd effect from childhood PCV13 immunization, which already reached around 65% in late 2012 [32]. Thus, we could not estimate PPV23 effectiveness against pneumococcal pneumonia in this study. As for cardiovascular complications, pneumococcal polysaccharide vaccination significantly reduced the risk of acute coronary events by 17% (95% CI 3–29%) on meta-analysis [33]. However, PPV23 did not show significant effectiveness in preventing cardiovascular complications in this study. Two points need to be considered in this study. First, most cardiovascular events were acute exacerbations of heart failure (95.4%), so most post-flu acute coronary events might be missed without clinical suspicion. Second, we only evaluated VE against post-flu cardiovascular events, not for pneumonia-related cardiovascular events.

The primary strength of this study was that vaccination records were reliably identified using national immunization registry data of the KCDC. Laboratory tests and clinical data collection were conducted following the standardized HIMM surveillance protocol. On the other hand, there were some limitations to this study. First, PCR-based influenza viral subtyping was not carried out for all laboratory-confirmed influenza cases. Second, as reported previously, some complicated influenza cases might not present typical ILI, so they may have been missed [34]. Third, in H3N2 virus dominant seasons, the low number of events limited the statistical power to evaluate the VE against acute exacerbation of chronic lung and heart diseases. Finally, late exacerbation (cases occurring at ≥4 weeks from ILI) of cardiopulmonary disease was not considered in this study.

## Conclusions

Influenza vaccination was effective in preventing pneumonia only during vaccine-matched seasons with good effectiveness against circulating influenza viruses. In addition, influenza vaccination showed a cardio-protective effect during a vaccine-matched A/H1N1-dominant season.

## Supporting information

S1 Table Crude influenza vaccine effectiveness (VE) against pneumonia and acute exacerbation of cardiopulmonary disease. (DOCX)

S2 Table Crude influenza vaccine effectiveness (VE) against hospitalization and 30-day mortality. (DOCX)

S3 Table Crude vaccine effectiveness (VE) of 23-valent pneumococcal polysaccharide vaccine against pneumonia and acute exacerbation of cardiopulmonary disease. (DOCX)

S4 Table Crude vaccine effectiveness (VE) of 23-valent pneumococcal polysaccharide vaccine against hospitalization and 30-day mortality. (DOCX)

## Abbreviations

ILI: influenza-like illness
LTCF: long-term care facility
PPV23: 23-valent pneumococcal polysaccharide vaccine
PCV13: 13-valent pneumococcal conjugate vaccine
AE: acute exacerbation
ICU: intensive care unit.
