# Supplementary material for: Effectiveness of influenza and pneumococcal polysaccharide vaccines against influenza-related outcomes including pneumonia and acute exacerbation of cardiopulmonary diseases: Analysis by dominant viral subtype and vaccine matching
Source: PLoS One. 2018 Dec 6;13(12):e0207918. doi: 10.1371/journal.pone.0207918 (PMC6283593; doi:10.1371/journal.pone.0207918)
Supplement: S1 Table — (DOCX) [file pone.0207918.s001.docx]

S1 Table. Crude Influenza Vaccine Effectiveness (VE) against Pneumonia and Acute Exacerbation of Cardiopulmonary Disease

| Season |  | Pneumonia | | AE of chronic airway disease | | AE of chronic heart disease | |
| --- | --- | --- | --- | --- | --- | --- | --- |
| 2014-2015 season | Crude VE (%) | 18 (-34 to 50) | | -78 (-579 to 53) | | 51 (-42 to 83) | |
|  | Cases, No. (events/total) | Vaccinated  42/349 | Non-vaccinated  33/231 | Vaccinated  8/349 | Non-vaccinated  3/231 | Vaccinated  6/349 | Non-vaccinated  8/231 |
| 2015-2016 season | Crude VE | 41 (16 to 58) | | -12 (-131 to 46) | | 78 (45 to 91) | |
|  | Cases, No. (events/total) | Vaccinated  64/476 | Non-vaccinated  96/463 | Vaccinated  16/476 | Non-vaccinated  14/463 | Vaccinated  6/476 | Non-vaccinated  25/463 |
| 2016-2017 Season | Crude VE | 53 (28 to 70) | | -161 (-1939 to 67) | | 23 (-115 to 73) | |
|  | Cases, No. (events/total) | Vaccinated  93/477 | Non-vaccinated  42/123 | Vaccinated  10/477 | Non-vaccinated  1/123 | Vaccinated  15/477 | Non-vaccinated  5/123 |
| Overall | Crude VE | 33 (15 to 46) | | -19 (-112 to 33) | | 57 (28 to 74) | |
|  | Cases, No. (events/total) | Vaccinated  199/1302 | Non-vaccinated  171/817 | Vaccinated  34/1302 | Non-vaccinated  18/817 | Vaccinated  27/1302 | Non-vaccinated  38/817 |
