# Supplementary material for: Effectiveness of influenza and pneumococcal polysaccharide vaccines against influenza-related outcomes including pneumonia and acute exacerbation of cardiopulmonary diseases: Analysis by dominant viral subtype and vaccine matching
Source: PLoS One. 2018 Dec 6;13(12):e0207918. doi: 10.1371/journal.pone.0207918 (PMC6283593; doi:10.1371/journal.pone.0207918)
Supplement: S2 Table — (DOCX) [file pone.0207918.s002.docx]

S2 Table. Crude Influenza Vaccine Effectiveness (VE) against Hospitalization and 30-day mortality

| Season |  | Hospitalization | | 30-day mortality | |
| --- | --- | --- | --- | --- | --- |
| 2014-2015 season | Crude VE (%) | 29 (-1 to 50) | | 85 (48 to 96) | |
|  | Cases, No. (events/total) | Vaccinated  98/349 | Non-vaccinated  82/231 | Vaccinated  3/349 | Non-vaccinated  13/231 |
| 2015-2016 season | Crude VE | 42 (24 to 55) | | 75 (42 to 89) | |
|  | Cases, No. (events/total) | Vaccinated  169/476 | Non-vaccinated  225/463 | Vaccinated  7/476 | Non-vaccinated  26/463 |
| 2016-2017 Season | Crude VE | 52 (27 to 68) | | 18 (-128 to 71) | |
|  | Cases, No. (events/total) | Vaccinated  226/477 | Non-vaccinated  80/123 | Vaccinated  16/477 | Non-vaccinated  5/123 |
| Overall | Crude VE | 32 (19 to 43) | | 64 (41 to 78) | |
|  | Cases, No. (events/total) | Vaccinated  493/1302 | Non-vaccinated  387/817 | Vaccinated  26/1302 | Non-vaccinated  44/817 |
