# Supplementary material for: Effectiveness of influenza and pneumococcal polysaccharide vaccines against influenza-related outcomes including pneumonia and acute exacerbation of cardiopulmonary diseases: Analysis by dominant viral subtype and vaccine matching
Source: PLoS One. 2018 Dec 6;13(12):e0207918. doi: 10.1371/journal.pone.0207918 (PMC6283593; doi:10.1371/journal.pone.0207918)
Supplement: S3 Table — (DOCX) [file pone.0207918.s003.docx]

S3 Table. Crude Vaccine Effectiveness (VE) of 23-valent Pneumococcal Polysaccharide Vaccine against Pneumonia and Acute Exacerbation of Cardiopulmonary Disease

| Season |  | Pneumonia | | AE of chronic airway disease | | AE of chronic heart disease | |
| --- | --- | --- | --- | --- | --- | --- | --- |
| 2014-2015 season | Crude VE (%) | 30 (-24 to 61) | | 7 (-257 to 75) | | -39 (-323 to 54) | |
|  | Cases, No. (events/total) | Vaccinated  17/166 | Non-vaccinated  58/414 | Vaccinated  3/166 | Non-vaccinated  8/414 | Vaccinated  5/166 | Non-vaccinated  9/414 |
| 2015-2016 season | Crude VE | -14 (-63 to 20) | | -80 (-274 to 13) | | 71 (16 to 90) | |
|  | Cases, No. (events/total) | Vaccinated  57/311 | Non-vaccinated  103/628 | Vaccinated  14/311 | Non-vaccinated  16/628 | Vaccinated  4/311 | Non-vaccinated  27/628 |
| 2016-2017 Season | Crude VE | 18 (-22 to 45) | | 9 (-215 to 74) | | 3 (-147 to 62) | |
|  | Cases, No. (events/total) | Vaccinated  84/394 | Non-vaccinated  51/206 | Vaccinated  7/394 | Non-vaccinated  4/206 | Vaccinated  13/394 | Non-vaccinated  7/206 |
| Overall | Crude VE | -8 (-36 to 14) | | -24 (-115 to 29) | | 27 (-22 to 57) | |
|  | Cases, No. (events/total) | Vaccinated  158/871 | Non-vaccinated  212/1248 | Vaccinated  24/871 | Non-vaccinated  28/1248 | Vaccinated  22/871 | Non-vaccinated  43/1248 |
