# Supplementary material for: Effectiveness of influenza and pneumococcal polysaccharide vaccines against influenza-related outcomes including pneumonia and acute exacerbation of cardiopulmonary diseases: Analysis by dominant viral subtype and vaccine matching
Source: PLoS One. 2018 Dec 6;13(12):e0207918. doi: 10.1371/journal.pone.0207918 (PMC6283593; doi:10.1371/journal.pone.0207918)
Supplement: S4 Table — (DOCX) [file pone.0207918.s004.docx]

S4 Table. Crude Vaccine Effectiveness (VE) of 23-valent Pneumococcal Polysaccharide Vaccine against Hospitalization and 30-day mortality

| Season |  | Hospitalization | | 30-day mortality | |
| --- | --- | --- | --- | --- | --- |
| 2014-2015 season | Crude VE (%) | 38 (6 to 59) | | 43 (-102 to 84) | |
|  | Cases, No. (events/total) | Vaccinated  40/166 | Non-vaccinated  140/414 | Vaccinated  3/166 | Non-vaccinated  13/414 |
| 2015-2016 season | Crude VE | 17 (-9 to 37) | | 25 (-63 to 66) | |
|  | Cases, No. (events/total) | Vaccinated  121/311 | Non-vaccinated  273/628 | Vaccinated  9/311 | Non-vaccinated  24/628 |
| 2016-2017 Season | Crude VE | 26 (-5 to 47) | | -5 (-164 to 58) | |
|  | Cases, No. (events/total) | Vaccinated  191/394 | Non-vaccinated  115/206 | Vaccinated  14/394 | Non-vaccinated  7/206 |
| Overall | Crude VE | 8 (-10 to 22) | | 16 (-38 to 49) | |
|  | Cases, No. (events/total) | Vaccinated  352/871 | Non-vaccinated  528/1248 | Vaccinated  26/871 | Non-vaccinated  44/1248 |
